# Intercellular mitochondrial transfer alleviates pyroptosis in dental pulp damage

**DOI:** 10.1111/cpr.13442

**Published:** 2023-04-21

**Authors:** Konghuai Wang, Lu Zhou, Hanqing Mao, Jiayi Liu, Zhi Chen, Lu Zhang

**Affiliations:** ^1^ The State Key Laboratory Breeding Base of Basic Science of Stomatology (Hubei‐MOST) & Key Laboratory of Oral Biomedicine Ministry of Education, School & Hospital of Stomatology Wuhan University Wuhan China; ^2^ Department of Endodontics, School and Hospital of Stomatology Wuhan University Wuhan China

## Abstract

Mitochondrial transfer is emerging as a promising therapeutic strategy for tissue repair, but whether it protects against pulpitis remains unclear. Here, we show that hyperactivated nucleotide‐binding domain and leucine‐rich repeat protein3 (NLRP3) inflammasomes with pyroptotic cell death was present in pulpitis tissues, especially in the odontoblast layer, and mitochondrial oxidative stress (OS) was involved in driving this NLRP3 inflammasome‐induced pathology. Using bone marrow mesenchymal stem cells (BMSCs) as mitochondrial donor cells, we demonstrated that BMSCs could donate their mitochondria to odontoblasts via tunnelling nanotubes (TNTs) and, thus, reduce mitochondrial OS and the consequent NLRP3 inflammasome‐induced pyroptosis in odontoblasts. These protective effects of BMSCs were mostly blocked by inhibitors of the mitochondrial function or TNT formation. In terms of the mechanism of action, TNF‐α secreted from pyroptotic odontoblasts activates NF‐κB signalling in BMSCs via the paracrine pathway, thereby promoting the TNT formation in BMSCs and enhancing mitochondrial transfer efficiency. Inhibitions of NF‐κB signalling and TNF‐α secretion in BMSCs suppressed their mitochondrial donation capacity and TNT formation. Collectively, these findings demonstrated that TNT‐mediated mitochondrial transfer is a potential protective mechanism of BMSCs under stress conditions, suggesting a new therapeutic strategy of mitochondrial transfer for dental pulp repair.

## Graphical abstract

LPS + ATP induced mitochondrial dysfunction and subsequent NLRP3 activation and pyroptosis in mDPC6T cells. mDPC6T cells‐secreted TNF‐α as a major factor in promoting TNT‐mediated mitochondrial transfer during the coculture of mBMSCs with mDPC6T cells and confirmed that the TNT formation was NF‐κB‐dependent. Importantly, mitochondria from mBMSCs are selectively transferred to injured mDPC6T cells, which protects mDPC6T cells from injury.

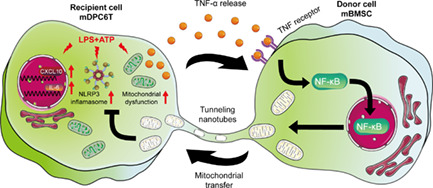

## 1 INTRODUCTION

Odontoblasts have been demonstrated to be the main functioning cells for dental pulp tissue repair.^1^ These highly specialized cells are arranged along the dentin–pulp interface and connected with each other through tight junctions, thus forming the front line of defence against external stimuli.^2^ It is, therefore, crucial to preserve odontoblast functionality and survival in the reparative process after pulp injury. Mitochondria are the central coordinators of energy metabolism, and alterations in their function lead to cellular oxidative stress (OS) and pyroptosis.^3^ Our previous study showed that mitochondrial damage in odontoblasts occurred in early pulpitis, leading to overproduction of mitochondrial reactive oxygen species (mtROS) and activation of nucleotide‐binding domain and leucine‐rich repeat protein3 (NLRP3) inflammasome‐induced pyroptosis.^4^ These effects eventually result in the structural disruption of the dentin‐pulp complex, which exacerbates pulpitis pathology.^5^ Together, mitochondrial damage and subsequent pyroptosis of odontoblasts are critical early events responsible for dental pulp injury and the progression of the disease.

Recently, mitochondrial transfer has appeared as an emerging therapeutic strategy as it can restore the bioenergetic requirements of injured cells.^6^, ^7^ As active mitochondrial donor cells, BMSCs are considered to be highly promising because of their abundant sources and multipotent differentiation and anti‐inflammatory properties.^8^ Several studies have discovered that BMSCs can donate their mitochondria to injured cells via TNTs, actin‐based intercellular channels that allow for direct communication between adjacent cells.^9^ The injured cells thus acquire functional mitochondria and regain their respiratory function and capacity for oxidative metabolism.^10^ Moreover, TNTs allow for bidirectional or unidirectional transfer of mitochondria in different cell types and contribute to the treatment of various inflammatory disorders, including neuroinflammation, retinal dysfunction and osteoarthritis.^11^, ^12^, ^13^, ^14^ However, whether BMSCs can provide mitochondria to odontoblasts and whether transferred mitochondria can rescue oxidative damage and pyroptosis in odontoblasts are still unclear.

The protective effects of BMSCs are linked to their capacity to migrate to the site of damaged tissue and to reduce oxidative damage, increase mitochondrial number, promote cell survival and inhibit pyroptosis locally.^11^, ^15^ Mechanistically, damage to tissues releases intracellular damage‐associated molecular patterns (DAMPs) to further amplify the inflammation process.^16^ Subsequently, the inflammatory factors released from injured cells could stimulate the mitochondrial biogenesis and TNT formation in BMSCs, thereby enhancing the mitochondrial transfer and rescue ability of BMSCs to injured cells.^17^ However, the conclusive mechanistic studies responsible for mitochondrial transfer under inflammatory conditions are still lacking.

In this study, we found that mitochondrial OS promotes NLRP3 inflammasome activation and pyroptosis in odontoblasts, which is the major cause of pulpitis. Furthermore, we confirmed that odontoblasts could receive BMSC‐derived mitochondria through TNTs, thereby reducing mitochondrial dysfunction and NLRP3 inflammasome‐induced pyroptosis. In further exploring the underlying mechanisms, we found that NF‐κB signalling is involved in the TNT formation. TNF‐α secreted by pyroptotic odontoblasts promoted the TNT formation in BMSCs via NF‐κB signalling, resulting in an increase in the mitochondrial transfer. This study furthers our understanding of TNT‐mediated mitochondrial transfer under stress conditions and advances its potential use for new therapeutic approaches in dental pulp repair.

## 2 MATERIALS AND METHODS

### 2.1 Human dental pulp tissue samples

All the dental pulp tissues were obtained as discarded biological samples from the School and Hospital of Stomatology, Wuhan University, following the approval of Institutional Ethics Medical Committee of Wuhan University (2019 LUNSHENZI(A48)). Normal pulps from teeth extracted for various reasons served as a control group (*n* = 31). Inflamed pulps extracted from patients diagnosed with irreversible pulpitis served as a pulpitis group (*n* = 65). All patients involved in this study provided a verbal informed consent and were diagnosed according to the published guidelines. In addition, patients receiving antibiotics or anti‐inflammatory drugs were excluded.

### 2.2 Cell culture

The odontoblast‐like cell line, mDPC6T cells, established in our previous study,^18^ was cultured in Dulbecco's Modified Eagle Medium (DMEM, Invitrogen) supplemented with 10% fetal bovine serum (FBS, Invitrogen) and antibiotics (#C0222, Beyotime Biotechnology) at 37°C in a humidified incubator with 5% CO_2_.

Mouse bone marrow mesenchymal stem cells (mBMSCs) used in this study were isolated from 4‐week‐old male C57BL/6 mice. Briefly, bone marrow was collected from femora and tibiae of mice and plated in tissue culture flasks containing DMEM supplemented with 10% FBS and antibiotics. After 2 days, we removed nonadherent cells in the supernatant and cultured adherent mBMSCs and used them after 4–10 passages.

### 2.3 Cell treatments

mDPC6T cells stimulated with LPS and ATP were utilized to simulate the pyroptosis during the pulpitis pathological process. In detail, mDPC6T cells were first primed with 1 μg/mL LPS (#LPS25, Sigma Aldrich) for 24 h, followed by treatment with 2 mM ATP (#HY‐B2176, MedChemExpress) for 24 h (referred to as LA‐mDPC6T). To inhibit mitochondrial OS, mDPC6T cells were pre‐treated with 5 μM MitoTEMPO (#HY‐112879, MedChemExpress) or 2 μM NAC (#HY‐B0215, MedChemExpress) for 4 h prior to stimulation with LPS and ATP. To inhibit mitochondrial transfer and tunnelling nanotube (TNT) formation, cells were pre‐treated with 1 μM cytochalasin B (HY‐16928, MedChemExpress) for 6 h to interfere polymerization and interaction of actin. For signalling pathway inhibition, cells were pre‐treated with 1 μM NF‐κB inhibitor Bay 11‐7082 (#HY‐13453, MedChemExpress) or 5 μM IKKβ inhibitor ML120B (#HY‐15473, MedChemExpress) for 4 h before experiments. To generate the mBMSCs without the mitochondrial function (ρ^o^mBMSCs), cells were cultured in a medium additioned with 1 μg/mL ethidium bromide (15585011, Invitrogen), 100 μg/mL pyruvic acid (HY‐Y0781, MedChemExpress) and 50 μg/mL uridine (HY‐B1449, MedChemExpress) for at least 1 month. For the TNF‐α treatment, the mBMSCs were treated with different concentrations of mouse TNF‐α (#HY‐P7090, MedChemExpress) for 24 h.

### 2.4 Coculture experiments and cell labelling

Direct coculture was performed with mDPC6T cells and mBMSCs at a density of 2 × 10^4^ cells/cm^2^ each (ratio 1:1) in DMEM. To explore the inhibitory effect of mBMSCs on pyroptosis, LPS‐primed mDPC6T cells were subjected to direct coculture with mBMSCs for 24 h in the culture medium supplemented with ATP (referred to as LA‐mDPC6T + mBMSC). To investigate mitochondrial transfer, mBMSCs were transfected with CellLight Mitochondria‐RFP (#C10601, Invitrogen) or stained with MitoAPC (#MT12, Dojindo) to label mitochondria according to the manufacturer's protocol. To distinguish mDPC6T cells from mBMSCs in mixed cultures, mDPC6T cells were stained with CellTrace CFSE (#C34554, Invitrogen). For F‐actin staining, fixed and permeabilized cells were stained with Alexa Fluor Plus‐405 Phalloidin (#A30104, Invitrogen) or Actin Tracker Red‐555 Phalloidin (#C2203S, Beyotime Biotechnology). Meanwhile, nuclei were counterstained with Hoechst (#C1017, Beyotime Biotechnology). For live cell sorting, cells were labelled with fluorescence dyes, treated as indicated and processed to cell sorting without prior fixation. Fluorescence‐activated cell sorting (FACS) performed on a FACSAria III cell sorter (BD Biosciences) was used to isolate mDPC6T cells and mBMSCs according to the manufacturer's instructions.

Indirect coculture of mDPC6T cells and mBMSCs was established using a transwell system (Nest Biotechnology) with 0.4 μm pore size polyethylene terephthalate inserts. Cell treatments, labelling and staining methods were performed as described above. To determine the paracrine effects of the pyroptotic mDPC6T cells, mBMSCs were seeded in the lower chamber, and LPS‐primed mDPC6T cells were seeded in the upper chamber, and then the culture medium supplemented with ATP and neutralizing antibodies of anti‐TNF‐α (#AF‐410, R&D Systems), anti‐IL‐6 (#MAB406, R&D Systems) or anti‐CXCL1 (#MAB453, R&D Systems) were added to transwell. Protein expression and phenotypic changes in mBMSCs were analysed after 24 h of coculture.

### 2.5 Bioinformatic and transcriptomic analysis

Total RNA was extracted from the cells using TRIzol (#15596018, Invitrogen) according to the protocol provided by the manufacturer. A total quantity of 2 μg of RNA per sample from the mDPC6T, LA‐mDPC6T and LA‐mDPC6T + mBMSC (LA‐mDPC6T sorted after coculture with mBMSCs) was used for analysis. The quality of extracted RNA was assessed using the Agilent 2100 Bioanalyzer (Agilent Technologies). Complementary DNA (cDNA) libraries were synthesized with the TruSeq RNA Sample Preparation Kit v.2 (Illumina). Libraries were sequenced on an Illumina HiS Equation 4000 platform at the Beijing Genomics Institute (BGI). Filtering and quality controls were applied according to the standard procedure. GO and KEGG pathway analysis was performed with DAVID Bioinformatics Resources (http://david.abcc.ncifcrf.gov/). Gene set enrichment analysis (GSEA) was performed using the GSEA v4.2.3 software.

### 2.6 Real‐time quantitative PCR (qPCR)

Total RNA was extracted with the TRIzol reagent (#15596018, Invitrogen) according to the manufacturer's instructions. The genomic DNA was removed, and the total RNA was converted to cDNA using Hiscript III RT SuperMix (#R323, Vazyme). qPCR was performed with the ChamQ SYBR qPCR Master Mix (#Q311‐02, Vazyme) and monitored in real time with the CFX96 Real‐time System (Bio‐Rad). Data were normalized to the housekeeping gene β‐actin, and relative expression was evaluated using the 2^−ΔΔCt^ method. Primer sequences were designed and included in Appendix Table 1.

### 2.7 TNT formation and mitochondrial transfer analysis

CFSE‐labelled mDPC6T cells and MitoAPC‐labelled mBMSCs were subjected to coculture at a 1:1 ratio under indicated conditions. Then, the mixed cells were collected, and flow cytometry analysis (CytoFLEX, Beckman Coulter) was used to quantitatively determine the rate of mitochondrial transfer from mBMSCs to mDPC6T cells. Gating strategy used for CFSE‐labelled mDPC6T cells in cocultured cells and mitochondrial transfer rate analysis (Appendix Figure 3A).

CFSE‐labelled mDPC6T cells were subjected to coculture with Mitochondria‐RFP‐transfected mBMSCs under indicated conditions. Cells were subsequently fixed, permeabilized and stained by Alexa Fluor Plus‐405 Phalloidin. Transferred mitochondria and formed TNT were observed under the confocal microscope (FV1200, Olympus) or fluorescence microscope (IX83, Olympus). The percentage of TNT‐forming mBMSC was estimated by counting a total of 300 cells from random fields.

### 2.8 Immunoblotting analysis

Immunoblotting analysis was performed according to a previously described protocol.^4^ In brief, equal amounts (20 μg) of total protein were loaded onto 10% SDS‐polyacrylamide gels and separated by electrophoresis. Then, the proteins were electrophoretic transfer onto a PVDF membrane in tris‐glycine buffer. After blocking with 5% skimmed milk in tris‐buffered saline supplemented with 0.1% Tween‐20 (TBST), membranes were incubated overnight with primary antibody: anti‐NLRP3 (#ab263899, Abcam), anti‐caspase1 (#ab179515, Abcam), anti‐IL‐1β (#ab234437, Abcam), anti‐Tom20 (#ab186734, Abcam), anti‐Cytochrome C (#ab110325, Abcam), anti‐Pink1 (#ab23707, Abcam), anti‐Parkin (#ab179812, Abcam), anti‐LC3B (#ab192890, Abcam), anti‐FUNDC1 (#ab224722, Abcam), anti‐Atg5 (#ab108327, Abcam), anti‐Atg7 (#ab133528, Abcam), anti‐SQSTM1/p62 (#ab109012, Abcam), anti‐mtTFA (#ab252432, Abcam), anti‐p‐IKKα/β (#ab194528, Abcam), anti‐IKKβ (#A19606, ABclonal), anti‐p‐IκBα (#2859, Cell Signaling Technology), anti‐IκBα (#4814, Cell Signaling Technology), anti‐p‐p65 (#3033, Cell Signaling Technology), anti‐p65 (#8242, Cell Signaling Technology) and anti‐β‐actin (#PMK058M; Bioprimacy). The membrane was washed in TBST and then incubated with corresponding secondary antibodies (ABclonal) at room temperature for 1 h, followed by the addition of an enhanced chemiluminescence substrate (Invitrogen). The signals were visualized on the Odyssey FC Imaging System (LiCor, Lincoln). The final image was quantified by densitometry using the Image Studio Lite 5.2 (LiCor) software. β‐actin and Tom20 were used as the internal reference to normalized protein expression levels. The experiment was repeated at least three times.

### 2.9 Coimmunoprecipitation (CoIP) analysis

CoIP analysis was performed using the Pierce Co‐Immunoprecipitation Kit (#26149, Thermo Fisher Scientific) by following the manufacturer's instruction. In brief, antibodies of anti‐FUNDC1 (#ab224722, Abcam) and anti‐IgG (#ab6709, Abcam) were immobilized by amino link plus coupling resin. Cell lysates were precleared using the control agarose resin and then added to the spin column containing antibody‐coupled resin and incubated overnight at 4°C. The bound proteins were eluted by elution buffer, followed by immunoblotting analysis.

### 2.10 TMRM and MitoSOX assay

Mitochondrial membrane potential was measured using Tetramethylrhodamine methyl ester perchlorate (TMRM, #T668, Invitrogen), mitochondrial mass was measured using Mitogreen (#M7514, Invitrogen) and mitochondrial ROS (mtROS) was stained with MitoSOX Red mitochondrial superoxide indicator (MitoSOX, #M36008, Invitrogen). Briefly, cells grown on chamber slides were treated as indicated and then stained with 100 nM TMRM, 100 nM Mitogreen or 2 μM MitoSOX for 30 min protected from light. After washed with the medium to remove the extracellular fluorescent probe, images were captured under a confocal microscope (FV1200, Olympus). The excitation wavelength was 559 nm for MitoSOX and TMRM and 488 nm for Mitogreen. For quantitative analysis, cells were collected by trypsinization and determined by flow cytometry analysis (FACSCelesta, BD Biosciences). For the detection of mtROS in tissue, fresh dental pulp tissues were harvested and immediately incubated with 10 μM MitoSOX Red for 30 min protected from light. Then, the tissues were frozen in cryomolds in the Optimal Cutting Temperature (OCT) compound, and frozen sections were prepared with a cryostat. All tissue sections were stained with Hoechst for nuclei, and fluorescence of sections were observed by the confocal microscope. The final image was quantified by the ImageJ 4.2 (National Institutes of Health) software. The percentage of MitoSOX positive cells was estimated by counting a total of 300 cells from random fields.

### 2.11 Measurement of ATP

Cells were collected by lysis buffer provided in the ATP assay kit (#S0026, Beyotime). The sample lysates were then centrifuged, and the supernatants were harvested for the ATP content analysis. Total protein concentration in supernatants was determined using the BCA assay (#P0011, Beyotime) according to the manufacturer's protocol. Before the ATP analysis, supernatants were mixed with ATP detection solution, and then intensity readings were taken with a microplate spectrophotometer (Bio‐Rad). The amount of ATP was calculated based on ATP standards and normalized to the concentration of total protein in each sample.

### 2.12 Immunofluorescence

Tissue sections or cells were fixed in 4% paraformaldehyde for 10 min. After washed with phosphate‐buffered saline (PBS), the samples were permeabilized with 0.2% Triton X‐100 in citrate buffer and then blocked with 1% BSA (bovine serum albumin) for 1 h. Blocked samples were incubated overnight with primary antibodies: anti‐NLRP3 (#ab263899, Abcam), anti‐caspase1 (#sc‐56036, Santa Cruz Biotechnology), anti‐Tom20 (#ab283317, Abcam), anti‐Pink1 (#ab23707, Abcam), anti‐Parkin (#ab179812, Abcam), anti‐p65 (#8242, Cell Signalling Technology), followed by fluorescent dye‐conjugated IgG secondary antibody (Abkkine) for 1 h. Next, samples were incubated with Hoechst for 10 min and observed with a fluorescence microscope (IX83, Olympus). The final image was quantified by the ImageJ 4.2 software.

### 2.13 Fluorescence imaging‐based analysis of mitophagy

The cultured cells were incubated with 100 nM Lyso‐Tracker Green (#C1047S, Beyotime) for 30 min to label lysosome, followed by the addition of 100 nM MitoAPC (#MT12, Dojindo) and further incubation for 30 min to label mitochondria. After wash with PBS, images were captured under a confocal microscope (LSM880, Carl Zeiss), and colocalization of lysosome and mitochondria was determined using the ZEN 3.5 (Carl Zeiss) software that shows co‐staining with green and red fluorescences.

### 2.14 Enzyme‐linked immunosorbent assay (ELISA) and caspase1 activity assay

The level of IL‐1β in cell culture supernatant was measured with a mouse IL‐1β ELISA kit (#CME0015, 4A Biotech) according to the manufacturer's instructions. Briefly, samples were incubated overnight in a 96‐well plate coated with primary capture antibody. Samples were then incubated with primary detection antibody for 2 h, followed by secondary antibody conjugated to horseradish peroxidase (HRP) for 30 min. Samples were washed with PBS and then colorimetric HRP substrate TMB (3,3′,5,5′‐tetramethylbenzidine) was added. After developing for 20 min, the stop solution was added to stop the reaction. A microplate spectrophotometer (Bio‐Rad) was used to read absorbance at 450 nm. The media incubated without cells were used to determine background signals. The concentration of IL‐1β was determined using the standard solutions prepared in parallel.

Caspase1 activity was evaluated using the caspase1 activity assay kit (#C1101, Beyotime Biotechnology) according to the manufacturer's instructions. The substrate was Ac‐YVAD‐pNA (acetyl‐Tyr‐Val‐Ala‐Asp p‐nitroanilide). Caspase1 could catalyse Ac‐YVAD‐pNA into the formazan product p‐nitroaniline (pNA). In brief, samples were incubated with Ac‐YVAD‐pNA at 37°C for 1 h. The absorbance values of pNA at 405 nm were tested using a microplate spectrophotometer (Bio‐Rad), and caspase1 activity was determined using the pNA standard solutions prepared in parallel.

### 2.15 Fluorescence resonance energy transfer (FRET) analysis

Briefly, cells grown on chamber slides were treated as indicated, fixed in 4% paraformaldehyde for 10 min, permeabilized with 0.2% Triton X‐100 for 30 min, and blocked with 1% BSA for 1 h. After the removal of BSA, the primary antibodies of anti‐NLRP3 (#ab263899, Abcam) and anti‐Tom20 (#ab283317, Abcam) were added and incubated overnight at 4°C. After washed with PBS, the cells were incubated with goat anti‐rabbit Dylight 488 (#A23220, Abbkine) and goat anti‐mouse Dylight 549 (#A23310, Abbkine) for 1 h at room temperature. Nuclei were stained with Hoechst. FRET efficiency was determined using the acceptor photobleaching methodology using the Olympus fluoviewer 4.2 (Olympus) software. For acceptor photobleaching, the acceptor (Dylight 549) was consistently photobleached from 10% to 80% of reduction in the fluorescence intensity. Pre‐ and post‐bleach images were recorded for both the donor and the acceptor, and FRET efficiency was expressed as the increase in the FRET donor after bleaching the FRET acceptor. FRET images were further analysed using the PIX‐FRET plugins for ImageJ.^19^

### 2.16 Mitochondria cytoplasmic fractionation

The cells were collected in the mitochondrial separation reagent provided in the cell mitochondria isolation kit (#89874, Thermo Fisher Scientific) and homogenized with the Dounce homogenizer. The homogenate was centrifuged at 3000*g* for 10 min. Next, the supernatant was collected and further centrifuged at 11,000*g* for 10 min, thereby obtaining the pellet as mitochondria‐enriched fraction for the subsequent experiments.

### 2.17 Data analysis

Statistically significant differences were determined by Student's *t* test (for two groups) and one‐way analysis of variance (ANOVA) (for multiple groups), as needed, using the GraphPad Prism 9.0 (GraphPad Software). Pearson's correlation coefficient was used for correlation analyses. *p* < 0.05 was considered to be statistically significant (*p < 0.05, **p < 0.01 and ***p < 0.001). Error bars represent the mean value ± standard deviation (mean ± SD).

## 3 RESULTS

### 3.1 Mitochondrial OS and NLRP3 inflammasome‐mediated pyroptosis contributes to the pathogenesis of pulpitis

**FIGURE 1:**
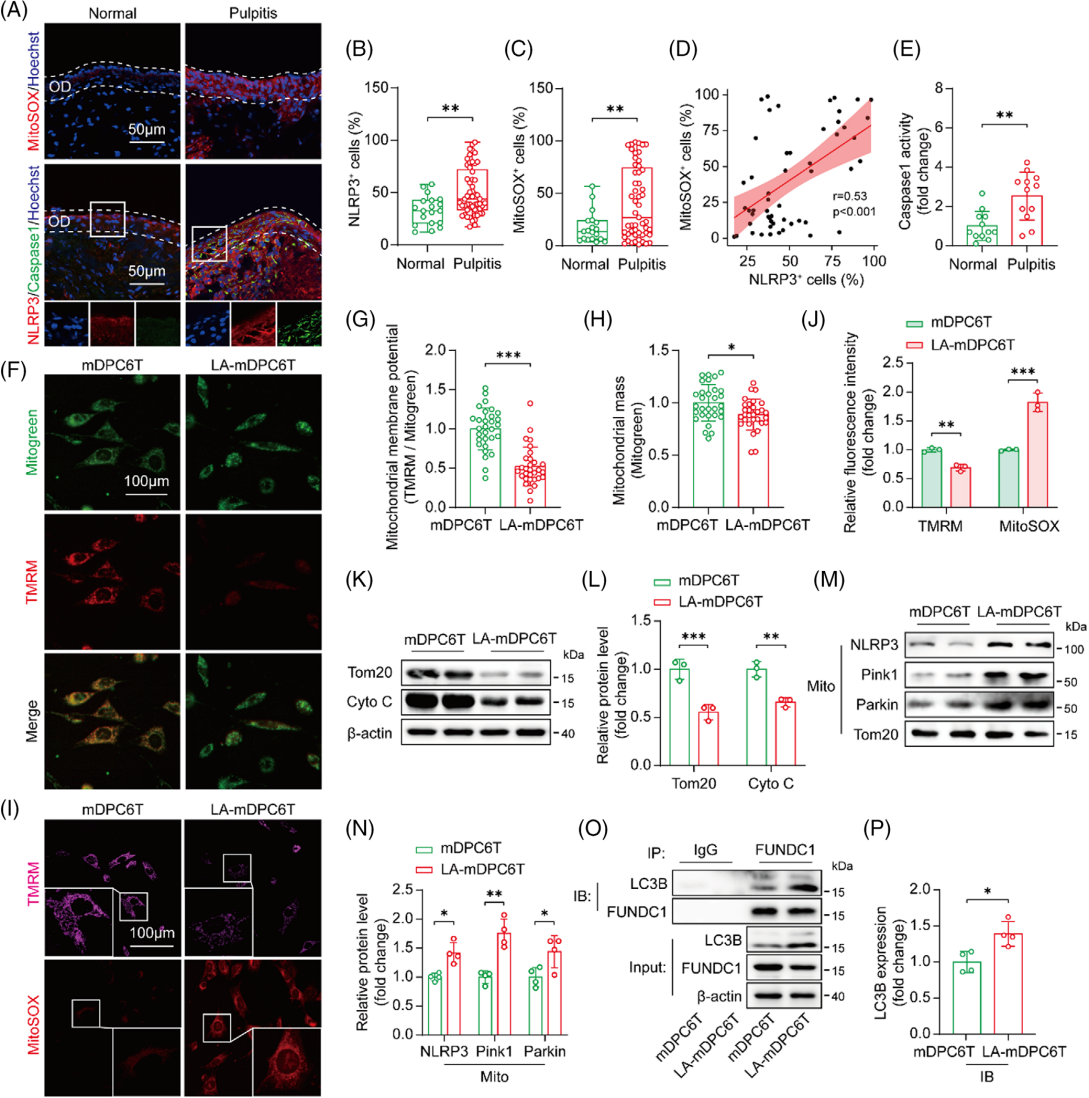
Mitochondrial OS mediates NLRP3 inflammasome activation and pyroptosis during pulpitis. (A) Immunofluorescence staining of NLRP3, caspase1 and MitoSOX in the pulp tissue. The nucleus was stained with Hoechst. OD: odontoblast layer. (B,C) Quantitative analysis of NLRP3‐ and MitoSOX‐positive cells in the dental pulp tissue from normal controls (*n* = 19) and pulpitis patients (*n* = 53). (D) The relationship between NLRP3‐ and MitoSOX‐positive cells in pulpitis tissue (*n* = 53). (E) Caspase1 activity in the dental pulp tissue from normal controls (*n* = 12) and pulpitis patients (*n* = 12). (F–H) Immunofluorescence staining and quantitative analysis of TMRM and Mitogreen in mDPC6T and LA‐mDPC6T cells (*n* = 30). (I,J) Immunofluorescence staining and flow cytometry analysis of TMRM and MitoSOX in mDPC6T and LA‐mDPC6T cells (*n* = 3). (K,L) Immunoblotting analysis of Tom20 and Cyto C protein expression in mDPC6T and LA‐mDPC6T cells (*n* = 3). (M,N) Immunoblotting analysis of NLRP3, Pink1 and Parkin protein expression in isolated mitochondria from mDPC6T and LA‐mDPC6T cells (*n* = 3). (O,P) Whole‐cell lysates from mDPC6T and LA‐mDPC6T cells were immunoprecipitated with FUNDC1 and IgG antibodies and then immunoblotted with the indicated antibodies, and the ratio between immunoprecipitated LC3B to FUNDC1 was quantified (*n* = 4). Data are displayed as the mean ± SD. Statistical significance was determined using Student's *t* test and one‐way analysis of variance (*p < 0.05; **p < 0.01; ***p < 0.001).

To investigate whether mitochondrial dysfunction and NLRP3 inflammasome signals are involved in pulpitis, dental pulp tissues were collected from patients with pulpitis and normal controls and analysed by immunofluorescence. The odontoblast layer stained positively for NLRP3 in pulpitis tissues compared with normal controls (Figure 1A,B). In addition, elevated MitoSOX levels were presented in pulpitis tissues, especially in the odontoblast layer, and this was positively correlated with NLRP3 expression (Figure 1A,C,D). Caspase1 expression in the odontoblast layer and its enzymatic activity were significantly increased in pulpitis tissues (Figure 1A,E).

mDPC6T cells (an odontoblast‐like cell line) stimulated with LPS and ATP were utilized to simulate odontoblast pyroptosis in the pathological process of pulpitis. In detail, mDPC6T cells were primed with 1 μg/mL LPS for 24 h, followed by treatment with 2 mM ATP for 24 h (LA‐mDPC6T). mDPC6T cells without any stimulation were denoted as the control (mDPC6T). LPS + ATP stimulation led to a loss of mitochondrial membrane potential (Figure 1F,G,I,J), reduction in mitochondrial mass (Figure 1F,H) and higher mtROS generation (Figure 1I,J). Tom20 and Cyto C are commonly used as mitochondrial markers to reflect mitochondrial mass.^20^ Immunoblotting analysis revealed that Tom20 and Cyto C protein expression levels were decreased in LA‐mDPC6T cells (Figure 1K,L), indicating a decrease in the mitochondrial mass. We speculate that this loss of mitochondrial mass may be due to the activation of mitophagy. FUNDC1 and the Pink1/Parkin pathway are known to function in regulating mitophagy. Immunoblotting analysis and immunofluorescence staining indicated that LPS + ATP stimulation enhanced the mitochondrial localization of Pink1 and Parkin (Figure 1M,N; Appendix Figure 1A). CoIP analysis showed that the interaction between FUNDC1 and LC3B was increased in LA‐mDPC6T cells (Figure 1O,P). These results indicated the activation of mitophagy in LA‐mDPC6T cells, which may account for the decreased mitochondrial mass. Treatment with the general antioxidant *N*‐acety‐l‐cysteine (NAC) or the mitochondrial‐targeted antioxidant MitoTEMPO significantly inhibited mitophagy in LA‐mDPC6T cells, as reflected by decreased mitochondrial colocalization with lysosomes (Appendix Figure 1B,C). Likewise, the autophagic flux in LA‐mDPC6T cells was blocked by NAC or MitoTEMPO treatment, as reflected by decreased protein expression of Atg5, Atg7 and LC3B‐II and increased expression of p62 (Appendix Figure 1D,E). The changes were accompanied by the restoration of mitochondrial mass in LA‐mDPC6T cells, which was assessed by immunoblotting for Tom20 and Cyto C (Appendix Figure 1F,G). Similarly, the enhanced mtROS generation and loss of mitochondrial membrane potential in LA‐mDPC6T cells were partially reversed by the NAC or MitoTEMPO treatment (Appendix Figure 1H,I). LPS + ATP‐induced NLRP3 inflammasome activation and pyroptosis were alleviated by NAC and MitoTEMPO treatment, as reflected by decreased protein expression of NLRP3, cleaved‐caspase1 and IL‐1β (Appendix Figure 1J,K) and decreased mRNA expression of IL‐6 and CXCL10 (Appendix Figure 1L). Recent studies have revealed that the mitochondrial localization of NLRP3 is essential for activating the NLRP3 inflammasome.^21^, ^22^ Consistent with our data, immunoblotting of NLRP3 expression in isolated mitochondria was increased by LPS + ATP stimulation (Figure 1J,K). FRET analysis was performed to further assess the mitochondrial localization of NLRP3 (Appendix Figure 2A). FRET analysis revealed that the LPS + ATP treatment facilitated NLRP3 translocation to mitochondria, and this process was abolished by NAC or MitoTEMPO (Appendix Figure 2B,C). These data demonstrate that mitochondrial OS, NLRP3 inflammasome activation and pyroptosis are present in pulpitis and suggest that improving mitochondrial homeostasis and rescuing mitochondrial mass could alleviate these phenomena in pulpitis.

### 3.2 Mitochondria from mBMSCs are selectively transferred to injured mDPC6T cells

**FIGURE 2:**
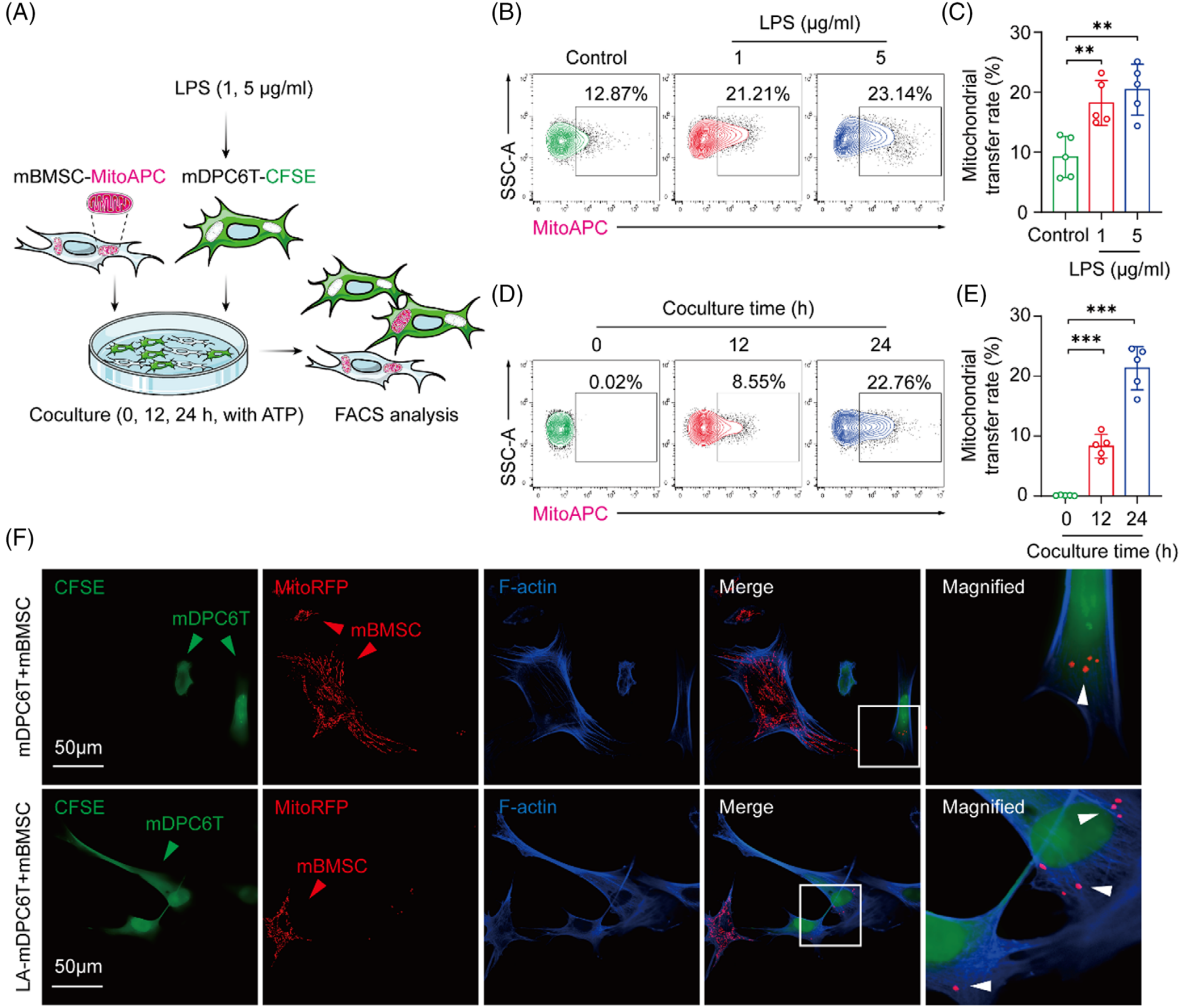
Mitochondria from mBMSCs are transferred to mDPC6T cells and the transfer rate is enhanced under stressed conditions. (A) Schematic representation of the coculture experimental design for the detection of mitochondrial transfer. CellTrace CFSE‐labelled mDPC6T cells (mDPC6T‐CFSE) were primed with LPS (1 or 5 μg/mL) for 24 h, followed by incubation with ATP (2 mM) for different times (0, 12 or 24 h). MitoAPC‐labelled mBMSCs (mBMSC‐MitoAPC) were added to mDPC6T cells in the ATP treatment step. The mitochondrial transfer rate was determined by flow cytometry. (B,C) mDPC6T cells were primed with LPS (1 and 5 μg/mL) for 24 h, followed by incubation with both ATP (2 mM) and mBMSCs for 24 h. mDPC6T cells without LPS or ATP treatment were denoted as the control. The mitochondrial transfer rate from mBMSCs to mDPC6T cells was determined by flow cytometry (*n* = 5). (D,E) mDPC6T cells were primed with LPS (1 μg/mL) for 24 h, followed by incubation with both ATP (2 mM) and mBMSCs for different times (0, 12 or 24 h). The mitochondrial transfer rate from mBMSCs to mDPC6T cells was determined by flow cytometry (*n* = 5). (F) Immunofluorescence staining of mitochondrial transfer from mBMSC‐MitoRFP to mDPC6T‐CFSE under stressed or normal conditions. The white arrowheads indicate the mitochondria from mBMSCs (red arrowheads) within mDPC6T cells (green arrowheads). Data are displayed as the mean ± SD. Statistical significance was determined using one‐way analysis of variance (**p < 0.01; ***p < 0.001).

BMSCs are the active mitochondrial donor cells for various cell types.^23^ To explore whether BMSCs could donate their mitochondria to mDPC6T cells, mBMSCs were subjected to coculture with healthy mDPC6T cells (control) or injured mDPC6T cells (LPS + ATP stimulation). In brief, CellTrace CFSE‐labelled mDPC6T cells (mDPC6T‐CFSE) were primed with LPS, followed by coculture with MitoAPC‐labelled mBMSCs (mBMSC‐MitoAPC) in the culture medium supplemented with ATP. Subsequently, the total cells were harvested and subjected to flow cytometry analysis (Figure 2A). After 24 h of coculture, the flow cytometry analysis showed a low mitochondrial transfer rate from mBMSCs to healthy mDPC6T cells, while injured mDPC6T cells received a significantly higher number of mitochondria from mBMSCs (Figure 2B,C), indicating that mBMSCs prefer to donate their mitochondria to injured mDPC6T cells rather than healthy mDPC6T cells. Moreover, the mitochondrial transfer rate was similar at low (1 μg/mL) and high (5 μg/mL) LPS concentrations. We next investigated the effects of varying the coculture time on the mitochondrial transfer rate at low LPS concentration. Flow cytometry analysis showed a time‐dependent increase in the mitochondrial transfer rate (Figure 2D,E). In addition, treatment with ATP alone did not significantly increase mitochondrial transfer from mBMSCs to mDPC6T cells (Appendix Figure 3B,C). We then expressed CellLight Mitochondria‐RFP in mBMSCs (mBMSC‐RFP) to further confirm intercellular mitochondrial transfer. RFP‐labelled mitochondria from mBMSCs were detected in several of the (LA‐)mDPC6T‐CFSE samples (Figure 2F). Collectively, these results indicate that mBMSCs have the capability of selectively donating their mitochondria to injured mDPC6T cells.

### 3.3 mBMSCs inhibit the mitochondrial OS and pyroptosis in mDPC6T cells through mitochondrial transfer

**FIGURE 3:**
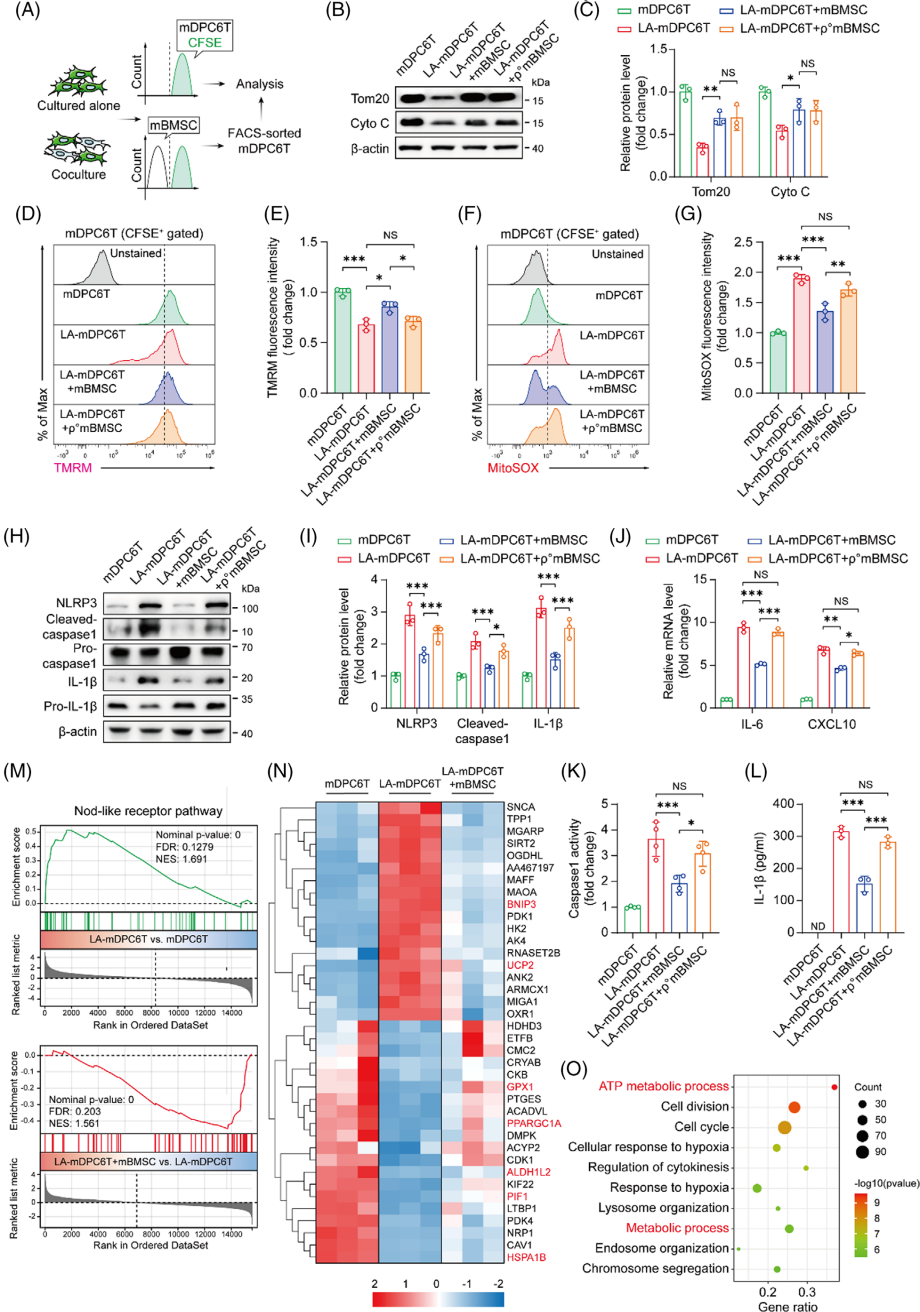
Mitochondrial transfer of mBMSCs protects mDPC6T cells from mitochondrial dysfunction, NLRP3 inflammasome activation and pyroptosis. (A) Schematic representation of the strategy to capture mDPC6T cells in cocultured cells. mDPC6T‐CFSE cells were subjected to coculture with mBMSCs, followed by cell sorting using FACS. CFSE‐positive cells were collected and then used for further analysis. (B,C) Immunoblotting analysis of Tom20 and Cyto C protein expression in mDPC6T cells and LA‐mDPC6T cells alone or cocultured with (ρ^o^)mBMSCs (*n* = 3). (D–G) Flow cytometry analysis of TMRM and MitoSOX in mDPC6T cells and LA‐mDPC6T cells alone or cocultured with (ρ^o^)mBMSCs (*n* = 3). (H,I) Immunoblotting analysis of NLRP3, caspase1 and IL‐1β protein expression (*n* = 3), (J) qPCR analysis of IL‐6 and CXCL10 mRNA expression (*n* = 3) and (K) caspase1 activity in mDPC6T cells and LA‐mDPC6T cells alone or cocultured with (ρ^o^)mBMSCs (*n* = 4). (L) ELISA analysis of IL‐1β levels in the cell culture medium of mDPC6T cells and LA‐mDPC6T cells alone or cocultured with (ρ^o^)mBMSCs (*n* = 3). (M) GSEA results of the NLR pathway in LA‐mDPC6T versus mDPC6T cells and LA‐mDPC6T + mBMSCs versus LA‐mDPC6T cells. (N) Heatmap showing the expression of metabolic genes in mDPC6T cells and LA‐mDPC6T cells alone or cocultured with mBMSCs. (O) GO enrichment analysis of the differentially expressed genes (DEGs) between LA‐mDPC6T cells and LA‐mDPC6T + mBMSCs. Data are displayed as the mean ± SD. Statistical significance was determined using one‐way analysis of variance (*p < 0.05; **p < 0.01; ***p < 0.001).

Next, we investigated whether mBMSCs could rescue injured mDPC6T cells through the transfer of mitochondria. We generated mBMSCs without the mitochondrial function (ρ^o^mBMSCs) via ethidium bromide (EtBr)‐mediated depletion of mtDNA. The depletion of mtDNA in ρ^o^mBMSCs, as confirmed by immunoblotting analysis (Appendix Figure 3D,E), resulted in reduced ND1 mRNA expression (Appendix Figure 3F) and decreased cellular ATP levels (Appendix Figure 3G). We collected LA‐mDPC6T cells in mixed cultures by cell sorting using FACS (Figure 3A). Immunoblotting analysis showed that Tom20 and Cyto C protein expression in LA‐mDPC6T cells was increased after coculture with mBMSCs or ρ^o^mBMSCs (Figure 3B,C), indicating the transfer of donor mitochondria to recipient cells. The transfer rate from mBMSCs to mDPC6T cells was similar to that from ρ^o^mBMSCs to mDPC6T cells (Appendix Figure 3H,I). The flow cytometry analysis revealed that only mBMSCs could restore the mitochondrial function in LA‐mDPC6T cells, and ρ^o^mBMSCs showed no significant impact on the mitochondrial function (Figure 3D–G). We then examined the effect of mBMSCs and ρ^o^mBMSCs on the NLRP3 inflammasome pathway and pyroptosis. NLRP3, cleaved‐caspase1 and IL‐1β protein expression in LA‐mDPC6T cells was inhibited after coculture with mBMSCs (Figure 3H,I). However, ρ^o^mBMSCs showed an inability to inhibit the expression of these proteins. Similar results were observed for the inhibition of IL‐6 and CXCL10 mRNA expression (Figure 3J), caspase1 activity (Figure 3K) and IL‐1β protein expression (Figure 3L). These results suggest that mBMSCs can rescue injured mDPC6T cells through the transfer of healthy mitochondria, rather than non‐functional mitochondria.

We performed RNA‐sequencing analysis to further confirm the protective effect of mBMSCs. The GSEA analysis showed that the NLR pathway was significantly enriched in the LA‐mDPC6T group compared to the mDPC6T group, but this result was reversed by coculture of LA‐mDPC6T cells with mBMSCs (Figure 3M). Moreover, by investigating the metabolic gene expression in LA‐mDPC6T cells, we found increased expression of genes involved in mitochondrial apoptosis (BNIP3 and UCP2). The expression levels of antioxidase (GPX1 and ALDH1L2) and mitochondrial metabolic genes (PPARGC1A, PIF1 and HSPA1B) were significantly decreased (Figure 3N). In contrast, coculture of LA‐mDPC6T cells with mBMSCs led to an opposite change in the expression of these genes (Figure 3N). GO enrichment analysis revealed that the ATP metabolic and metabolic processes were markedly enriched in the LA‐mDPC6T + mBMSC group compared to the LA‐mDPC6T group (Figure 3O). Together, these results demonstrate that the mitochondrial transfer of mBMSCs assisted in the rebalancing of mitochondrial redox homeostasis and inhibited NLRP3‐mediated pyroptosis in recipient cells.

### 3.4 TNT mediates mitochondrial transfer from mBMSCs to mDPC6T cells

**FIGURE 4:**
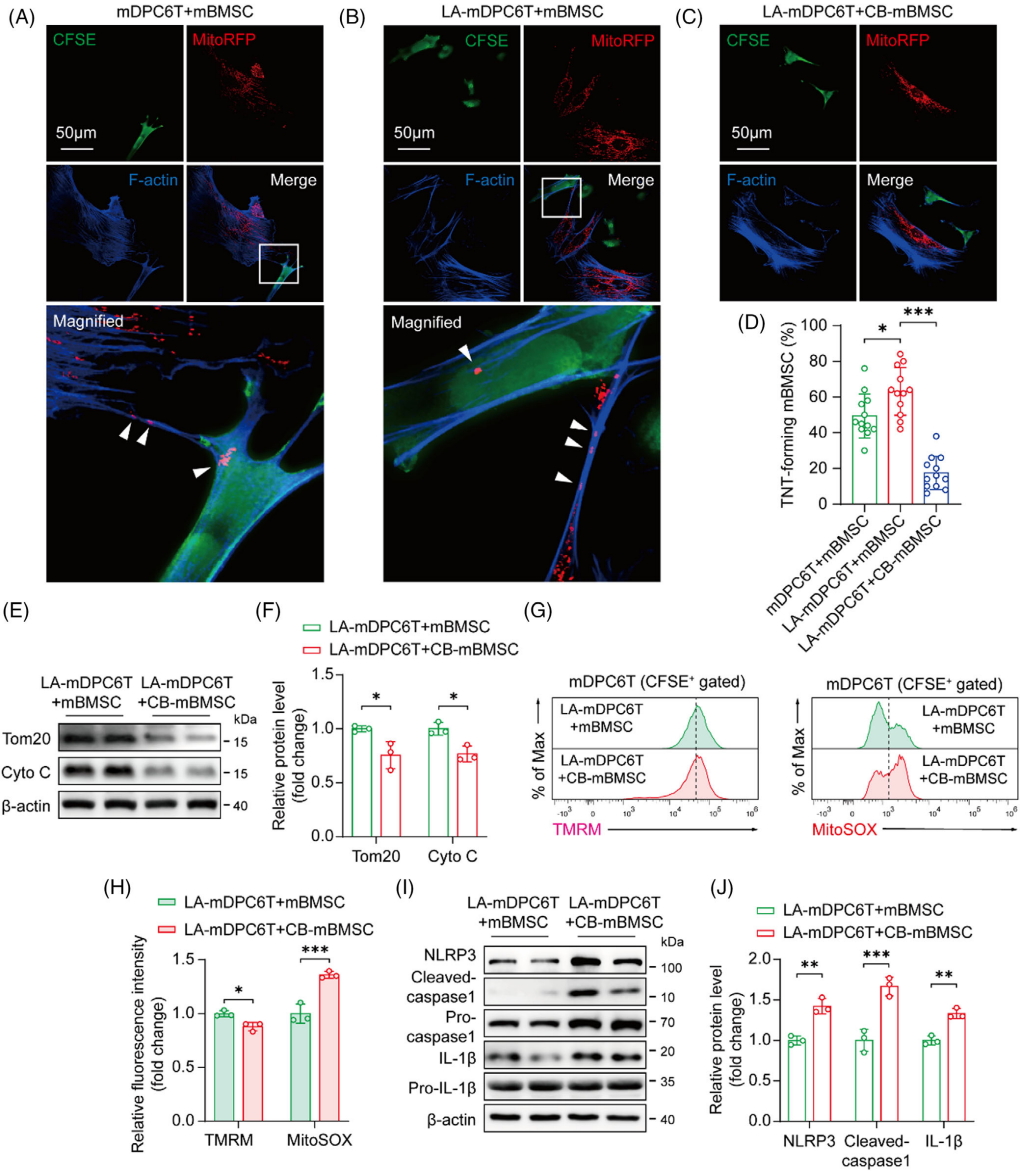
Tunnelling nanotube (TNT) mediates the mitochondrial transfer from mBMSCs to mDPC6T cells and protect mDPC6T cells from injury. Immunofluorescence staining of mitochondrial transfer between (A) mDPC6T cells and mBMSCs, (B) LA‐mDPC6T cells and mBMSCs, and (C) LA‐mDPC6T cells and CB‐mBMSCs. Mitochondria (arrowheads) from mBMSC‐RFP translocate along a TNT toward mDPC6T‐CFSE. (D) Quantitative analysis of the TNT‐forming rate of mBMSCs under the conditions described above (*n* = 12). (E,F) Immunoblotting analysis of Tom20 and Cyto C protein expression in LA‐mDPC6T cells cocultured with mBMSCs or CB‐mBMSCs (*n* = 3). (G,H) Flow cytometry analysis of TMRM and MitoSOX in LA‐mDPC6T cells cocultured with mBMSCs or CB‐mBMSCs (*n* = 3). (I,J) Immunoblotting analysis of NLRP3, caspase1 and IL‐1β protein expression in LA‐mDPC6T cells cocultured with mBMSCs or CB‐mBMSCs (*n* = 3). Data are displayed as the mean ± SD. Statistical significance was determined using one‐way analysis of variance (*p < 0.05; **p < 0.01; ***p < 0.001).

Mitochondrial transfer through TNTs is one of the mechanisms used by MSCs to repair tissue damage and to promote tissue regeneration.^14^ We next tested for TNT‐mediated transfer of mitochondria between mDPC6T cells and mBMSCs. mBMSC‐MitoRFP were cocultured with (LA‐)mDPC6T‐CFSE, and phalloidin was used to stain F‐actin in the TNTs. We observed that mBMSCs and (LA‐)mDPC6T cells physically connected via TNTs, which enabled the consecutive passage of mitochondria (Figure 4A,B). We also observed the colocalization of RFP‐labelled mitochondria within the TNTs. In addition, RFP‐labelled mitochondria were detected in mDPC6T cells, supporting mitochondrial transfer from mBMSCs to mDPC6T or LA‐mDPC6T cells via TNTs (Figure 4A,B). Pretreatment of mBMSCs with cytochalasin B (CB), an inhibitor of TNTs, significantly reduced the mitochondrial transfer from mBMSCs to LA‐mDPC6T cells (Figure 4C; Appendix Figure 3J,K). Interestingly, compared to mDPC6T cells, LA‐mDPC6T cells enhanced the TNT‐forming rate of mBMSCs, but this phenotype was completely suppressed by the CB pretreatment (Figure 4A–D). Immunoblotting analysis showed that pretreatment of mBMSCs with CB (CB‐mBMSC) reduced their ability to donate mitochondria (Figure 4E,F). To further evaluate the role of TNTs in mitochondrial transfer, we set up an indirect transwell coculture system, where the mBMSCs and mDPC6T cells were separated by a membrane with 0.4 μm pores that allowed secreted cytokines, free mitochondria and microvesicles to pass through but prevented direct physical contact via TNTs (Appendix Figure 4A). We found that physical contact but not microvesicle release or exocytosis was a prerequisite for mitochondrial transfer (Appendix Figure 4A). In direct coculture, pre‐treatment of mBMSCs with CB significantly inhibited its protective effect on the mitochondrial function, which was reflected in decreased TMRM and increased MitoSOX levels (Figure 4G,H). Similarly, the protective effect of mBMSCs against NLRP3‐mediated pyroptosis was also abolished by the CB pretreatment (Figure 4I,J; Appendix Figure 4B–D). These results confirm that TNTs participate in mitochondrial transfer and mediate the antipyroptotic function of mBMSCs.

### 3.5 TNF‐α activates the NF‐κB signalling in mBMSCs and promotes mitochondrial transfer

**FIGURE 5:**
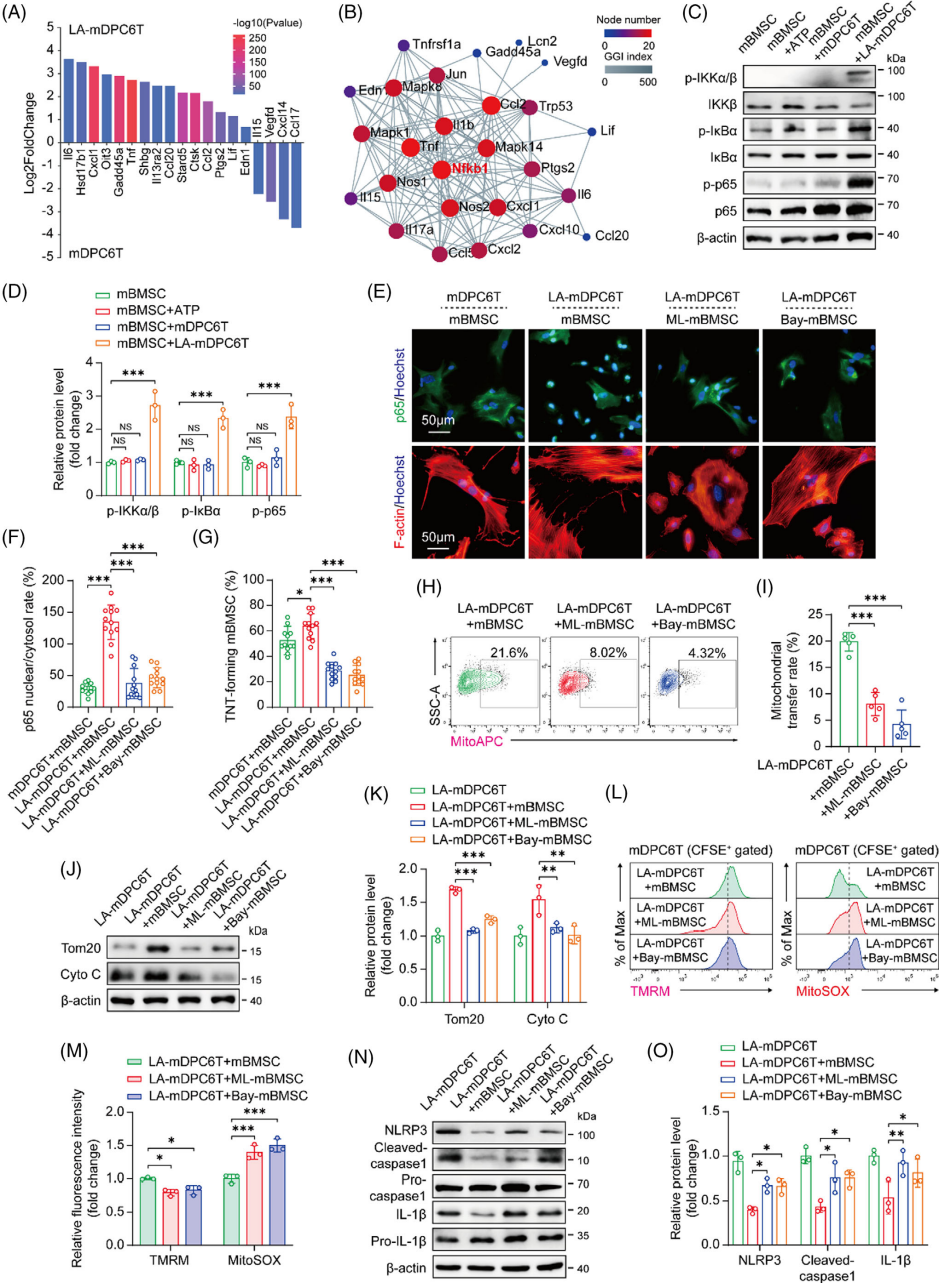
The NF‐κB pathway mediates tunnelling nanotube (TNT) formation and mitochondrial transfer from mBMSCs to mDPC6T cells. (A) RNA sequencing analysis of DEGs in LA‐mDPC6T cells compared to mDPC6T cells and (B) GGI interaction networks of these differentially expressed cytokines and their significantly interacting genes. (C,D) Immunoblotting analysis of NF‐κB pathway‐associated protein expression in mBMSCs, mBMSCs treated with ATP, and mBMSCs cocultured with mDPC6T or LA‐mDPC6T cells (*n* = 3). Coculture was performed in the transwell system as described in Appendix Figure 4A. (E) Immunofluorescence staining of p65 and TNT in mBMSCs under the indicated transwell conditions and (F,G) quantitative analysis of the p65 nuclear translocation rate and TNT‐forming rate (*n* = 12). (H,I) Flow cytometry analysis of mitochondrial transfer from mBMSCs to mDPC6T cells under indicated coculture conditions (*n* = 5). (J,K) Immunoblotting analysis of Tom20 and Cyto C protein expression in LA‐mDPC6T cells alone and LA‐mDPC6T cells cocultured with mBMSCs, ML‐mBMSCs or Bay‐mBMSCs (*n* = 3). (L,M) Flow cytometry analysis of TMRM and MitoSOX in LA‐mDPC6T cells cocultured with mBMSCs, ML‐mBMSCs or Bay‐mBMSCs (*n* = 3). (N,O) Immunoblotting analysis of NLRP3, caspase1 and IL‐1β protein expression in LA‐mDPC6T cells alone and LA‐mDPC6T cells cocultured with mBMSCs, ML‐mBMSCs or Bay‐mBMSCs (*n* = 3). Data are displayed as the mean ± SD. Statistical significance was determined using one‐way analysis of variance (*p < 0.05; **p < 0.01; ***p < 0.001).

Previous results have indicated that mBMSCs prefer to donate their mitochondria to injured mDPC6T cells. We next investigated the potential molecular mechanism underlying this phenomenon. The GO and KEGG pathway enrichment analyses revealed marked enrichment of the inflammatory response and cytokine–cytokine receptor interaction between the mDPC6T group and the LA‐mDPC6T group (Appendix Figure 5A). Therefore, we speculate that LA‐mDPC6T cells may secrete inflammatory cytokines, exerting paracrine effects on mBMSCs and then promoting mitochondrial transfer. RNA‐sequencing analysis of mDPC6T and LA‐mDPC6T cells identified several differentially expressed genes (DEGs) encoding inflammatory cytokines (Figure 5A), and the gene–gene interaction (GGI) network suggested that these genes interact with MAPK and NOS, with the most prominent interaction being with NF‐κB (Figure 5B). To determine whether these inflammatory cytokines secreted by LA‐mDPC6T cells can affect the NF‐κB pathway in mBMSCs, we utilized a transwell system. Coculture of mBMSCs with LA‐mDPC6T cells in transwell plates increased p‐IKKβ (phosphorylated IKKβ), p‐IκBα (phosphorylated IκBα) and p‐p65 (phosphorylated p65) protein expression in mBMSCs (Figure 5C,D). Coculture with mDPC6T cells or treatment with ATP alone did not change NF‐κB pathway‐associated protein expression in mBMSCs (Figure 5C,D). In addition, LA‐mDPC6T cells upregulated p65 nuclear translocation and TNT formation in mBMSCs under transwell (Figure 5E–G) or direct coculture conditions (Appendix Figure 5B–E). However, this upregulation was suppressed by pretreatment of mBMSCs with the NF‐κB inhibitor Bay 11‐7082 (Bay‐mBMSC) or the IKKβ inhibitor ML120B (ML‐mBMSC) (Figure 5E–G; Appendix Figure 5B–E). Bay‐mBMSCs and ML‐mBMSCs showed reduced mitochondrial donor capacity (Figure 5H–K; Appendix Figure 5B–E) and a lack of protective effects against mitochondrial dysfunction (Figure 5L,M) and NLRP3‐induced pyroptosis (Figure 5N,O; Appendix Figure 5F–H). These results confirmed that cytokines secreted by LA‐mDPC6T cells activated the NF‐κB pathway in mBMSCs, which promoted intercellular TNT formation and mitochondrial transfer.

We then aimed to identify the exact inflammatory factors responsible for activating the NF‐κB pathway in mBMSCs. Using neutralizing antibodies, we blocked TNF‐α, IL‐6 and CXCL1 in a transwell system and examined NF‐κB pathway‐associated protein expression in mBMSCs. Only inhibition of TNF‐α prevented upregulation of p‐IKKβ, p‐IκBα and p‐p65 protein expression in mBMSCs cocultured with LA‐mDPC6T cells (Appendix Figure 6A,B) and suppressed TNT formation (Appendix Figure 6C,D). Blocking TNF‐α also reduced the mitochondrial transfer from mBMSCs to mDPC6T cells during direct coculture (Appendix Figure 6E). Conversely, recombinant TNF‐α alone sufficed to induce NF‐κB protein expression levels similar to those of LA‐mDPC6T cells in mBMSCs (Appendix Figure 6F,G). Treatment of mBMSCs with TNF‐α alone upregulated TNT formation (Appendix Figure 6H,I). Moreover, the addition of TNF‐α to the culture medium during direct coculture promoted mitochondrial transfer from mBMSCs to mDPC6T cells (Appendix Figure 6J). Together, LPS + ATP induced mitochondrial dysfunction and subsequent NLRP3 activation and pyroptosis in mDPC6T cells, resulting in the production of a variety of inflammatory cytokines, such as IL‐1β and IL‐6. Among these inflammatory cytokines, we identified TNF‐α as a major factor in promoting TNT‐mediated mitochondrial transfer during the coculture of mBMSCs with mDPC6T cells and confirmed that TNT formation was NF‐κB‐dependent. Importantly, mitochondria from mBMSCs are selectively transferred to injured mDPC6T cells, which protects mDPC6T cells from injury.

## 4 DISCUSSION

With the progression of caries, odontoblasts are affected first because they are the first line of cells to come into contact with oral bacteria.^24^ The bacteria‐induced mitochondrial damage of odontoblasts is associated with the aberrant production of mtROS and is thought to contribute to the pathogenesis of pulpitis.^4^ Given the essential role of mitochondrial homeostasis in pulpitis, we postulated that delivery of heathy mitochondria to odontoblasts might compensate for mitochondrial damage and restore a basal metabolic capacity. In this study, we provide direct experimental evidence that mBMSCs can provide their mitochondria to injured mDPC6T cells through TNTs, thus preserving mitochondrial oxidative metabolism and bioenergetic requirements. Moreover, we revealed that the paracrine effect of mDPC6T cells on mBMSCs is the major driver of mitochondrial transfer.

Mitochondria are essential for energy metabolism and normal cellular function. Any mitochondrial abnormality can cause a bioenergetic crisis, eventually leading to inflammation and cell death.^25^, ^26^ Recent findings suggest that mtROS released from damaged mitochondria are the major trigger of NLRP3 inflammasome activation.^3^, ^21^ Consistently, our results showed that increased mtROS mediated NLRP3 inflammasome activation and subsequent pyroptosis of mDPC6T cells. In addition, mitochondrial damage facilitates the recruitment of NLRP3 to the mitochondria and may enhance its activation by allowing for efficient sensing of mtROS.^27^ Here, we found a similar phenomenon, in which inhibition of mtROS decreased NLRP3 mitochondrial localization and inflammasome activation. These findings provide new mechanistic links among NLRP3 inflammasome activation, mitochondrial dysfunction and pyroptosis during pulpitis.

Intercellular mitochondrial transfer has recently been described as a new mechanism to rescue mitochondrial function in injured cells.^28^, ^29^ In this study, we demonstrated that mBMSCs could donate their mitochondria to mDPC6T cells, thereby improving mitochondrial function and protecting mDPC6T cells from pyroptosis. RNA‐sequencing analysis further confirmed that mitochondria transferred from mBMSCs to mDPC6T cells may contribute to the mitochondrial biogenesis of mDPC6T cells. These results indicated that BMSCs could induce mitochondrial metabolic reprogramming in damaged cells and that mitochondrial donation could sustain the energy supply and redox balance.

Various routes are involved in mitochondrial transfer, such as TNT formation, microvesicle release, gap junctions and other routes of transfer.^23^ In this study, we revealed that TNTs are the main route of mitochondrial transfer between mBMSCs and mDPC6T cells. More interestingly, TNT formation in mBMSCs was increased in the pyroptosis model, and the mitochondrial transfer efficiency from mBMSCs to mDPC6T cells was also enhanced. The cells displaying numerous aberrancies and injuries are more permissive to mitochondrial transfer than their healthy cell counterparts.^28^ A variety of stress signals released by recipient cells are sensed by MSCs resulting in enhanced mitochondrial biogenesis and TNT formation by MSCs through retrograde signalling, thereby preparing MSCs for mitochondrial donation.^6^, ^10^ In addition, inflammatory factors released by cells suffering from OS and pyroptosis have also been postulated to trigger donation of mitochondria.^30^ Our results found that pyroptotic mDPC6T cells released TNF‐α that induced the TNT formation in mBMSCs via the NF‐κB pathway, which enhanced mitochondrial donation. The NF‐κB pathway has been shown to be involved in the TNT formation and stability by modulating actin interactions.^31^ In addition to NF‐κB, M‐Sec‐induced actin polymerization is an important initiating step of TNT formation.^32^ Following initiation, the expression of the actin regulatory protein Eps8, mammalian target of rapamycin (mTOR) and CDC42 appears necessary for the elongation of TNTs.^14^ Furthermore, Miro1, a Rho GTPase that helps mitochondrial movement along microtubules, is rate‐limiting for mitochondrial transfer.^33^ However, the exact regulatory mechanism of mitochondrial transfer and TNT formation remains unknown, and further experiments are required to investigate these cellular processes.

In conclusion, we revealed that mBMSCs promote reparative mechanisms in pulpitis through TNT‐mediated mitochondrial transfer. These findings highlight a potential clinical application of mitochondrial transfer and provide an important basis for the future development of mitochondria‐targeted therapy for dental pulp repair.

## AUTHOR CONTRIBUTIONS

Konghuai Wang carried out the experimental work, the collection and interpretation of data and drafted the manuscript; Lu Zhou participated in the study design, the collection and analysis of data and preparation of the manuscript; Hanqing Mao and Jiayi Liu carried out the study design, the analysis and interpretation of data and critically revised the manuscript; Zhi Chen and Lu Zhang conceived and designed the study and critically revised the manuscript. All authors have approved the final manuscript and have agreed to be accountable for all the aspects of the work.

## CONFLICT OF INTEREST STATEMENT

The authors declare that there is no conflict of interest regarding the publication of this article.

## Supporting information


**Data S1:** Supporting Information

## Data Availability

The data used to support the findings of this study are available from the corresponding authors upon request.
