# Supplementary material for: Intercellular mitochondrial transfer alleviates pyroptosis in dental pulp damage
Source: Cell Prolif. 2023 Apr 21;56(9):e13442. doi: 10.1111/cpr.13442 (PMC10472516; doi:10.1111/cpr.13442)
Supplement: Supplementary file 1 — Data S1: Supporting Information [file CPR-56-e13442-s001.docx]

**Intercellular mitochondrial transfer alleviates pyroptosis in dental pulp damage**

**Konghuai Wang^1^, Lu Zhou^1^, Hanqing Mao^1^, Jiayi Liu^1^, Zhi Chen^1, 2^, Lu Zhang^1, 2,^** *

^1^The State Key Laboratory Breeding Base of Basic Science of Stomatology (Hubei-MOST) & Key Laboratory of Oral Biomedicine Ministry of Education, School & Hospital of Stomatology, Wuhan University, Wuhan, China.

^2^Department of Endodontics, School and Hospital of Stomatology, Wuhan University, Wuhan, China.

* **Correspondence:**

L. Zhang, Department of Endodontics, School and Hospital of Stomatology, Wuhan University, LuoYu Road No.237, HongShan District, Wuhan, 430079, China. Email: luzhang2012@whu.edu.cn. Telephone: +86-27-8768-6208.

**Appendix Figures**


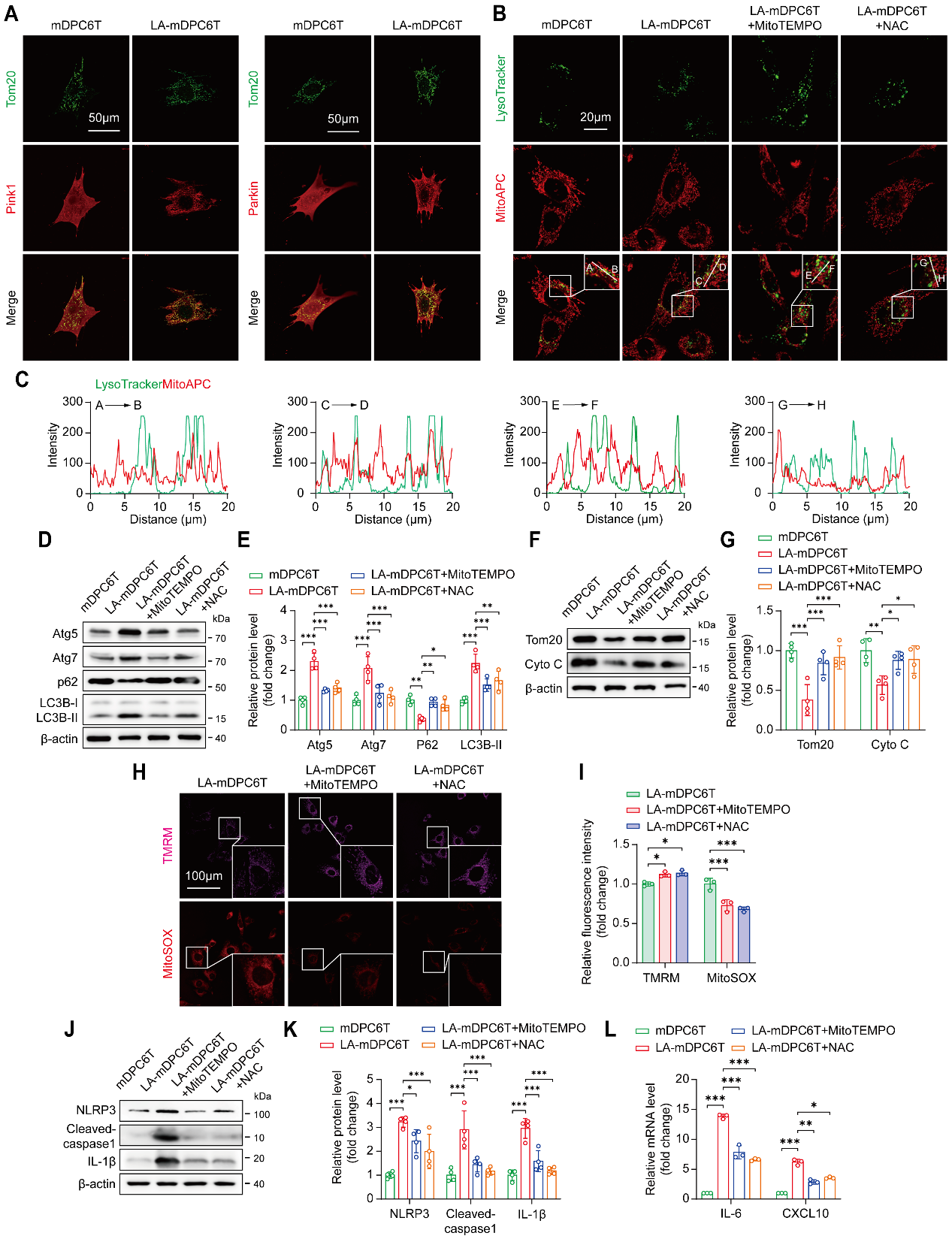


**Appendix Figure 1.** **(A)** Immunoﬂuorescence staining of Pink1/Parkin and Tom20 in mDPC6T and LA-mDPC6T cells. **(B)** Immunoﬂuorescence staining of LysoTracker and MitoAPC in mDPC6T cells, LA-mDPC6T cells, LA-mDPC6T+MitoTEMPO and LA-mDPC6T+NAC, and **(C)** fluorescence profiles along the corresponding white lines. **(D-G)** Immunoblotting analysis of Atg5, Atg7, p62, LC3B, Tom20 and Cyto C protein expression in mDPC6T cells, LA-mDPC6T cells, LA-mDPC6T+MitoTEMPO and LA-mDPC6T+NAC (n = 3). **(H)** Immunoﬂuorescence staining of MitoDeepRed and MitoSOX in LA-mDPC6T cells, LA-mDPC6T+MitoTEMPO and LA-mDPC6T+NAC, and **(I)** quantification by flow cytometry analysis (n = 3). **(J, K)** Immunoblotting analysis of NLRP3, caspase1 and IL-1β protein expression (n = 4) and **(L)** qPCR analysis of IL-6 and CXCL10 mRNA expression (n = 3) in mDPC6T cells, LA-mDPC6T cells, LA-mDPC6T+MitoTEMPO and LA-mDPC6T+NAC. Data are displayed as mean ± SD. Statistical significance was determined using one-way ANOVA (**P*<0.05; ***P*<0.01; ****P*<0.001).


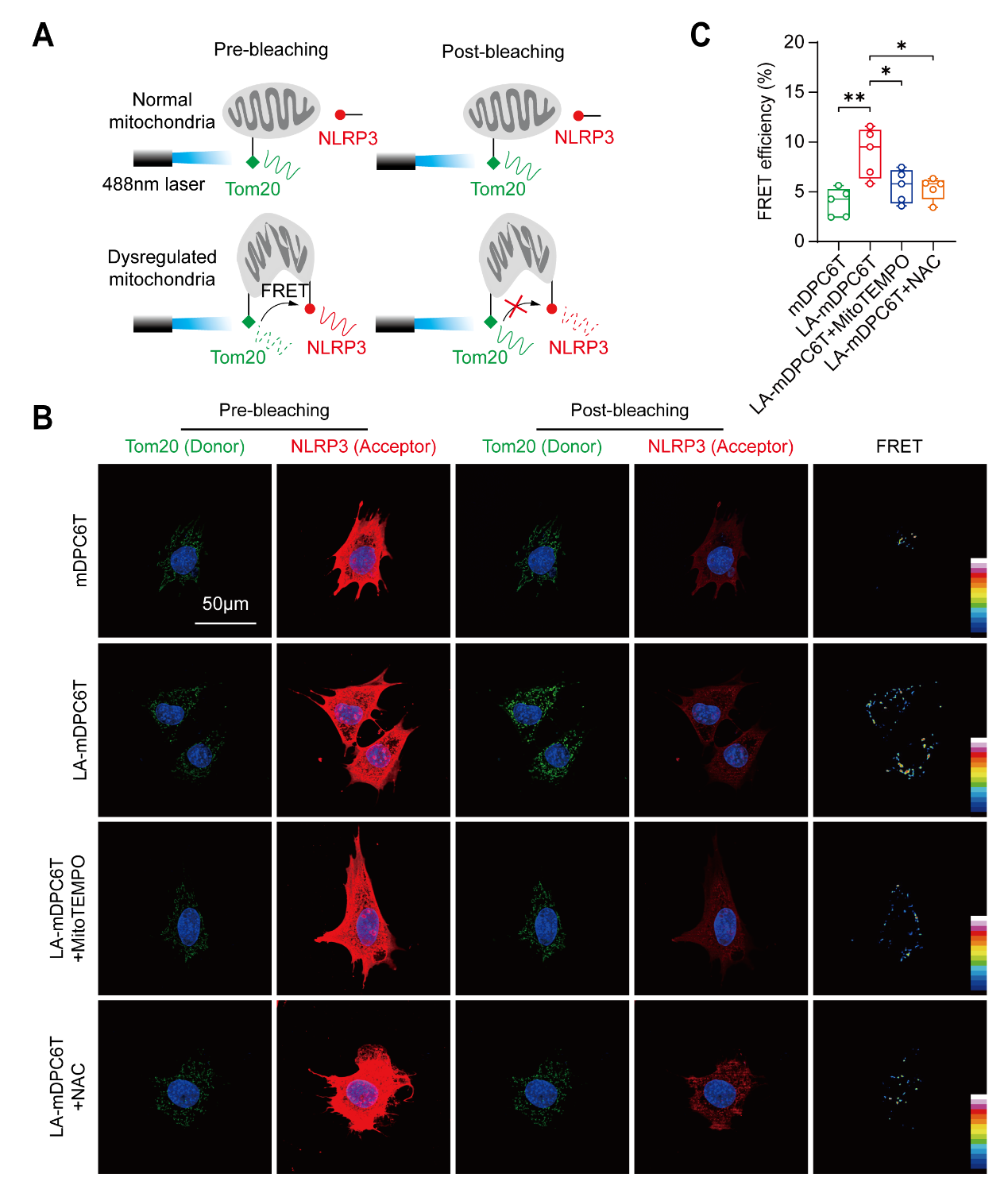


**Appendix Figure 2.** **(A)** Schematic depiction of the experimental design for the FRET analysis. FRET indicator contains Tom20 (green) as donor and NLRP3 (red) as acceptor. Following photo-bleaching (488nm laser) of the FRET acceptor, protein interaction is indicated by an increase in donor fluorescence. **(B, C)** Immunoﬂuorescence staining and FRET analysis for NLRP3 and Tom20 in mDPC6T cells, LA-mDPC6T cells, LA-mDPC6T+MitoTEMPO and LA-mDPC6T+NAC (n = 5). Statistical significance was determined using one-way ANOVA (**P*<0.05; ***P*<0.01).


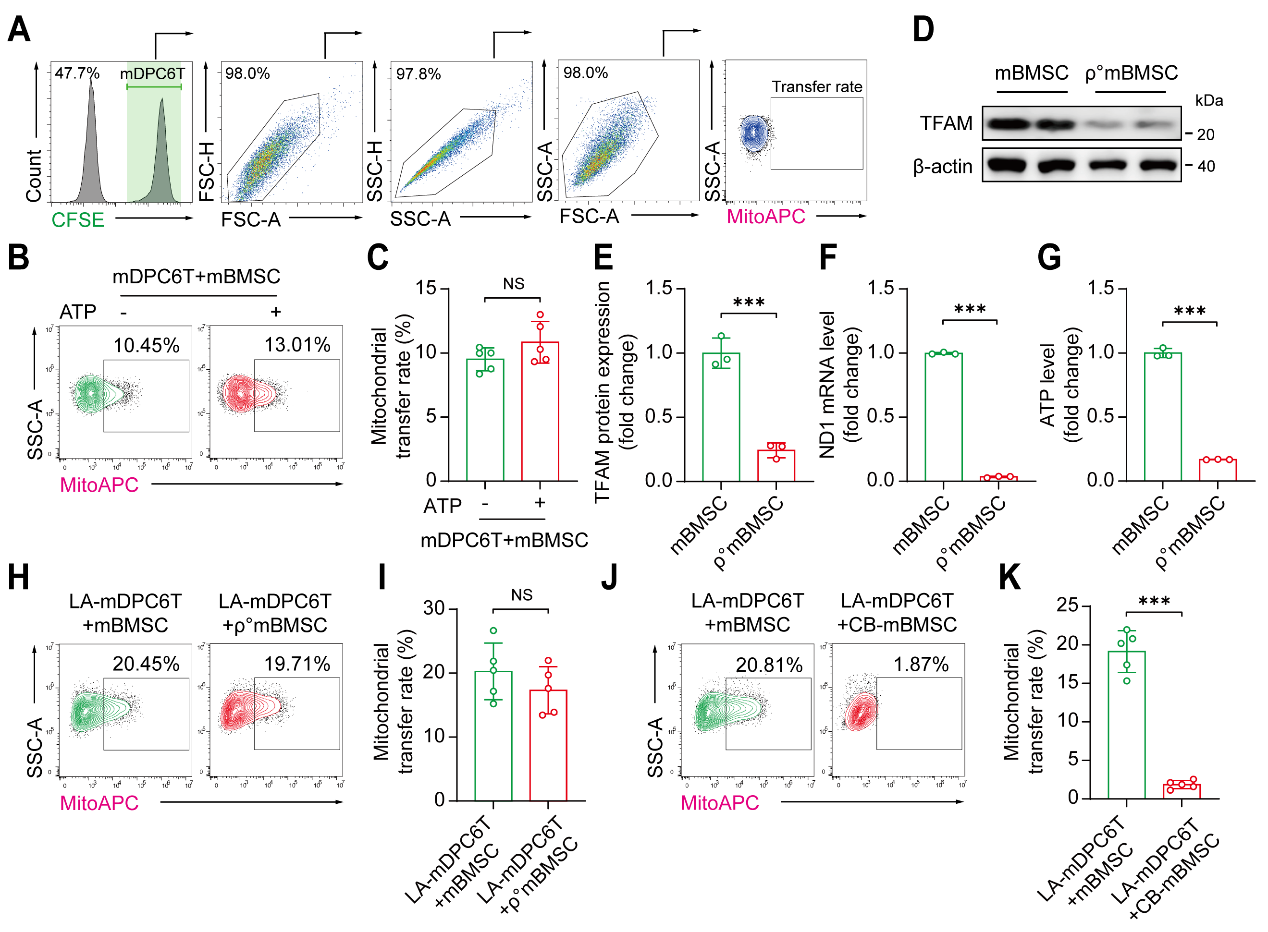


**Appendix Figure 3.** **(A)** Gating strategy to measure the rate of mitochondrial transfer from mBMSC-MitoAPC to mDPC6T-CFSE cells. **(B, C)** Flow cytometry analysis of mitochondrial transfer from mBMSCs to mDPC6T cells in the absence or presence of ATP (n = 5). **(D, E)** Immunoblotting analysis of TFAM protein expression (n = 3), **(F)** qPCR analysis of ND1 mRNA expression (n = 3) and **(G)** ATP level (n = 3) in mBMSCs and ρ^o^mBMSCs. **(H-K)** Flow cytometry analysis of mitochondrial transfer from mBMSCs to mDPC6T cells under indicated coculture condition (n = 5). Data are displayed as mean ± SD. Statistical significance was determined using one-way ANOVA (****P*<0.001). TFAM: mitochondrial transcription factor A; ND1: mitochondrially encoded NADH ubiquinone oxidoreductase core subunit 1.


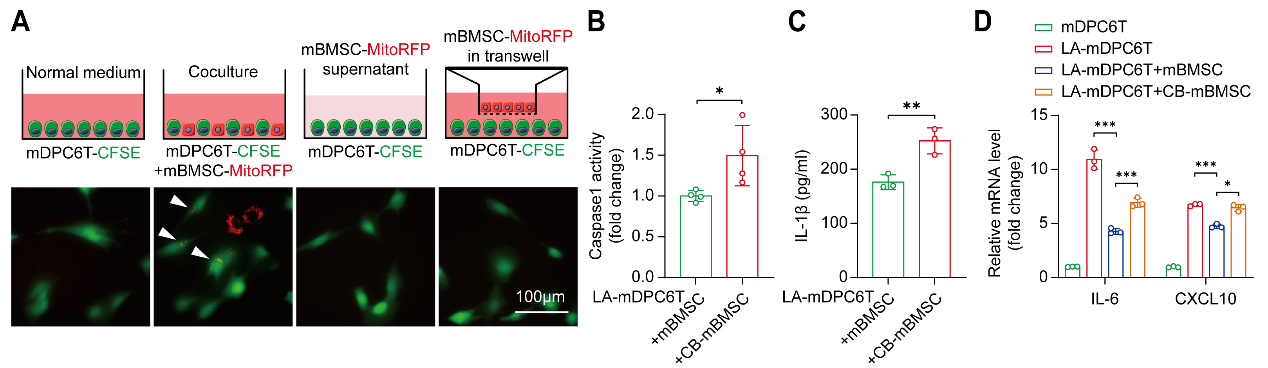


**Appendix Figure 4. (A)** Schematic depiction of the coculture experimental design using a transwell system. mDPC6T-CFSE cells on the lower transwell chamber were cocultured with normal medium, mBMSC-MitoRFP in direct contact, supernatant of mBMSC-MitoRFP or mBMSC-MitoRFP on the upper transwell chamber. The arrowheads indicate the mitochondria from mBMSCs within mDPC6T cells. **(B)** Caspase1 activity in LA-mDPC6T cells cocultured with mBMSCs or CB-mBMSCs (n = 4). **(C)** ELISA analysis of IL-1β level in the cell culture medium of LA-mDPC6T cells cocultured with mBMSCs or CB-mBMSCs (n = 3). **(D)** qPCR analysis of IL-6 and CXCL10 mRNA expression in mDPC6T cells and LA-mDPC6T cells alone or cocultured with (CB-)mBMSCs (n = 3). Data are displayed as mean ± SD. Statistical significance was determined using Student’s *t* test and one-way ANOVA (**P*<0.05; ***P*<0.01; ****P*<0.001).


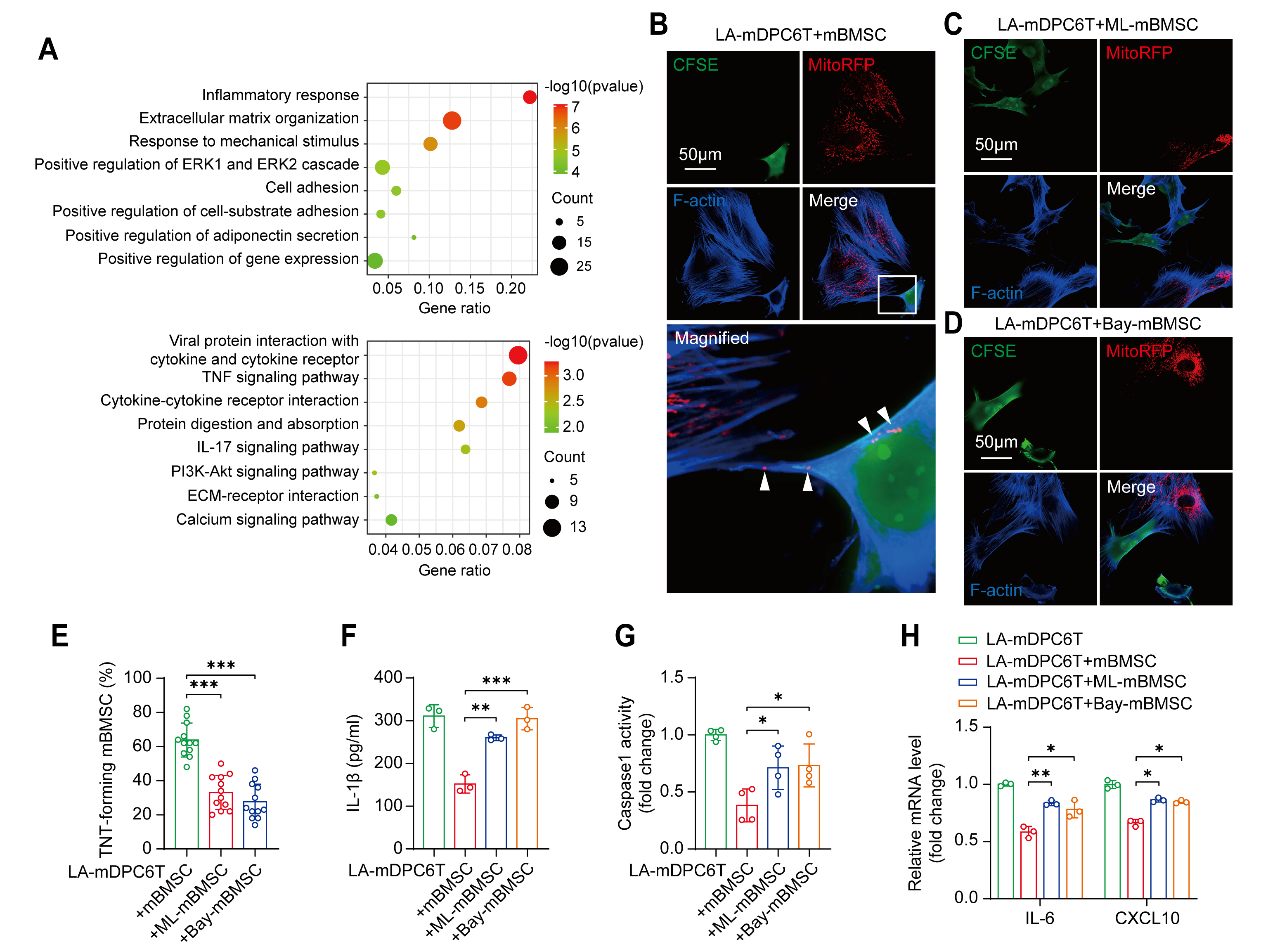


**Appendix Figure 5. (A)** The GO (top) and KEGG pathway (bottom) enrichment analysis of the DEGs between mDPC6T cells and LA-mDPC6T cells. Immunoﬂuorescence staining of mitochondrial transfer between **(B)** LA-mDPC6T cells and mBMSCs, **(C)** LA-mDPC6T cells and ML-mBMSCs, **(D)** LA-mDPC6T cells and Bay-mBMSCs. Mitochondria (arrowheads) from mBMSC-RFP translocate along a TNT toward mDPC6T-CFSE cells. **(E)** Quantitative analysis of TNT-forming rate of mBMSCs under the conditions as described above (n = 12). **(F)** ELISA analysis of IL-1β level in the cell culture medium of LA-mDPC6T cells alone and LA-mDPC6T cells cocultured with mBMSCs, ML-mBMSCs or Bay-mBMSCs (n = 3). **(G)** Caspase1 activity (n = 4) and **(H)** qPCR analysis of IL-6 and CXCL10 mRNA expression (n = 3) in LA-mDPC6T cells alone and LA-mDPC6T cells cocultured with mBMSCs, ML-mBMSCs or Bay-mBMSCs. Data are displayed as mean ± SD. Statistical significance was determined using one-way ANOVA (**P*<0.05; ***P*<0.01; ****P*<0.001).


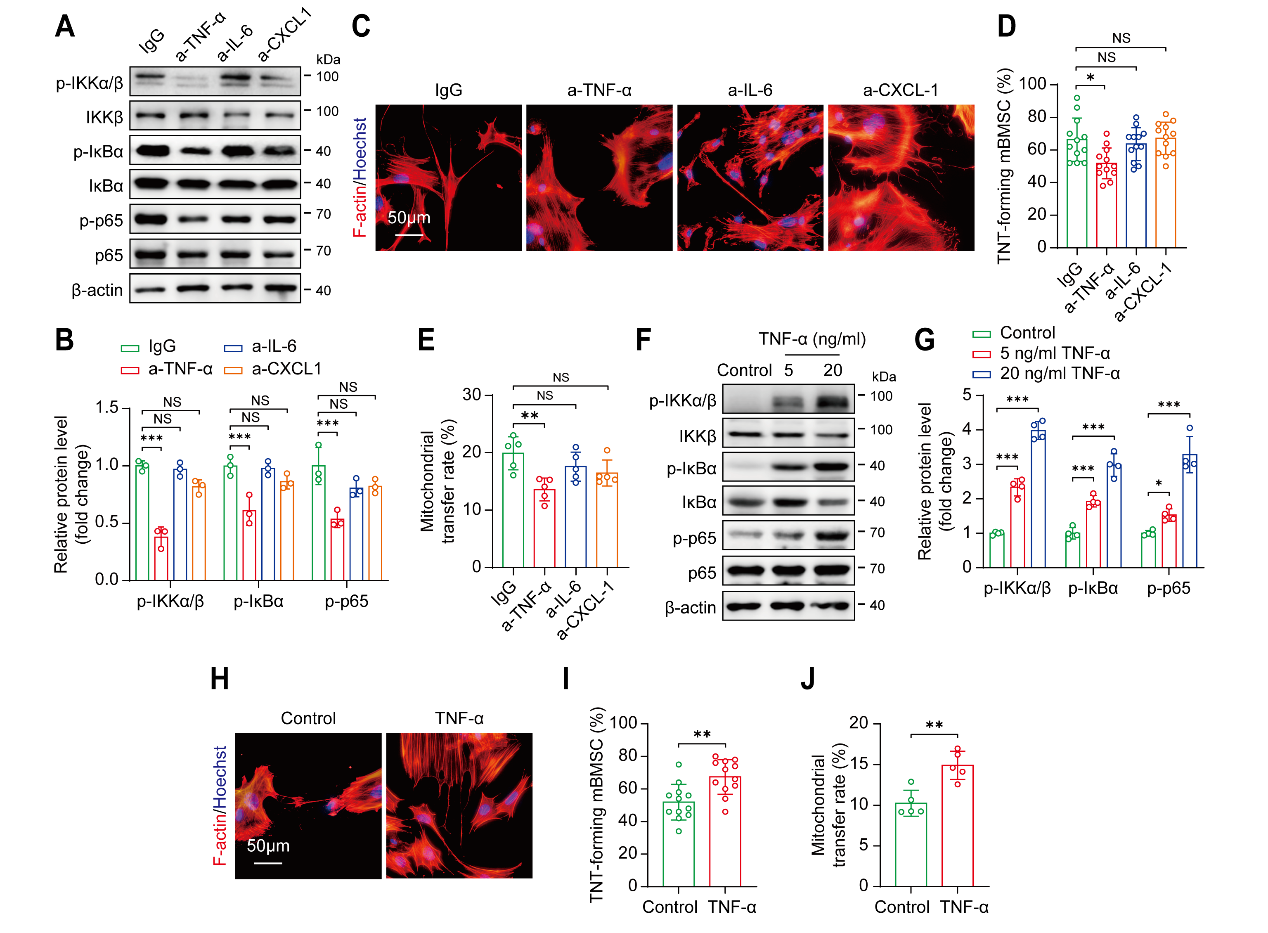


**Appendix Figure 6.** mBMSCs on the lower transwell chamber were cocultured with LA-mDPC6T cells on the upper transwell chamber in the presence of control IgG, a-TNF-α, a-IL-6 or a-CXCL1 neutralizing antibodies. **(A, B)** Immunoblotting analysis of NF-κB pathway-associated protein expression (n = 3), **(C)** immunoﬂuorescence staining of TNTs and **(D)** quantitative analysis of TNT-forming rate (n = 12) in mBMSCs under indicated transwell conditions. **(E)** Flow cytometry analysis of mitochondrial transfer from mBMSCs to mDPC6T cells in the presence of control IgG, a-TNF-α, a-IL-6 or a-CXCL1 neutralizing antibodies (n = 5). **(F, G)** Immunoblotting analysis of NF-κB pathway-associated protein expression in mBMSCs treated with or without TNF-α (n = 4). **(H)** Immunoﬂuorescence staining of TNTs and **(I)** quantitative analysis of TNT-forming rate (n = 12) in mBMSCs treated with or without TNF-α (20 ng/ml). **(J)** Flow cytometry analysis of mitochondrial transfer from mBMSCs to mDPC6T cells in the absence or presence of 20 ng/ml TNF-α (n = 5). Data are displayed as mean ± SD. Statistical significance was determined using Student’s *t* test and one-way ANOVA (**P*<0.05; ***P*<0.01; ****P*<0.001).

| **GENE** | **Forward (5’-3’)** | **Reverse (5’-3’)** |
| --- | --- | --- |
| *Il6* | TAGTCCTTCCTACCCCAATTTCC | TTGGTCCTTAGCCACTCCTTC |
| *Cxcl10* | TCCCTATGGCCCTCATTCTCA | CCAAGTGCTGCCGTCATTTTC |
| *mt-Nd1* | CTAGCAGAAACAAACCGGGC | CCGGCTGCGTATTCTACGTT |
| *Actb* | GTGACGTTGACATCCGTAAAGA | GCCGGACTCATCGTACTCC |

**Appendix Table 1. Primer sequences used for qPCR analysis in this study.**
